# Laser-Evoked Potentials in the Early Diagnosis of Diabetic Neuropathy and Their Association with Cardiovascular Autonomic Reflex Tests: A Retrospective Observational Study in Patients with Type 2 Diabetes

**DOI:** 10.3390/brainsci16040390

**Published:** 2026-03-31

**Authors:** Giovanni Umberto Borin, Marta Aventaggiato, Cristina Bittante, Vittorio Cacciatori, Alessia Segatti, Elisa Concon, Grazia Devigili, Enzo Bonora, Giacomo Zoppini, Giovanna Maddalena Squintani

**Affiliations:** 1 Neurology Unit, Department of Neuroscience, Biomedicine and Movement Sciences, Azienda Ospedaliera Universitaria Integrata of Verona, 37126 Verona, Italy; 2 Endocrinology and Diabetology, Rivoli Hospital, 10098 Rivoli, Italy; 3 Division of Metabolic Disease and Diabetology, University Hospital of Padua, 35128 Padua, Italy; 4 Endocrinology, Diabetology and Metabolic Disorders, University and Azienda Ospedaliera Universitaria Integrata of Verona, 37126 Verona, Italy; 5 IRCCS Istituto Neurologico “Carlo Besta”, 20133 Milan, Italy

**Keywords:** small-fibers neuropathy, laser-evoked potentials, T2DM, cardiovascular autonomic reflex tests, cardiovagal autonomic neuropathy

## Abstract

**Highlights:**

**What are the main findings?**

- Length-dependent LEP alterations detectable even in initial diabetic stages.
- Lack of correlation between LEP abnormalities and autonomic dysfunction.

**What are the implications of the main findings?**

- LEPs could be used as screening test in diabetic patients presenting peripheral neurological involvement.
- The lack of correlation between LEPs and CARTs may stem from the different types of fibers studied.

**Abstract:**

**Background:** Diabetic neuropathy manifests as symmetric distal and autonomic neuropathy, including cardiovagal dysfunction. Small-fiber involvement can occur, leading to neuropathic pain and dysautonomia. The diagnostic gold standard of these two conditions comprehends skin biopsy and cardiovascular autonomic reflex tests (CARTs), respectively. Non-invasive diagnostic tools, such as laser-evoked potentials (LEPs), show promise in detecting small-fiber damage, though correlations between LEP abnormalities and cardiovascular autonomic dysfunction remain poorly investigated. **Methods**: We retrospectively evaluated LEPs (from hands and feet stimulation) in 33 diabetic patients, comparing them to a cohort of 33 age-matched healthy subjects, to highlight any significant abnormalities in the diabetic cohort. We further analyzed the LEP results in T2DM cohort with clinical, laboratory variables and CARTs to explore potential correlations and to assess whether any association between LEPs and CARTs could be identified. **Results**: N2/P2 complex amplitude was significantly reduced in diabetic patients compared to healthy subjects, with greater involvement in the lower limbs. While no association between LEP abnormalities and abnormal CARTs was observed, LEP amplitude reductions were notably associated with elevated glycated hemoglobin levels and longer disease duration, which appeared to be the strongest predictor of LEP reduction. **Conclusions**: Our findings corroborated literature data regarding length-dependent LEP alterations detectable even in initial diabetic stages. The lack of correlation between LEP abnormalities and autonomic dysfunction may stem from the predominant involvement of C fibers in autonomic neuropathy, which are not adequately assessed by currently used LEPs.

## 1. Introduction

Neuropathy is a common complication of diabetes with an estimated lifetime prevalence exceeding 50% [1,2]. Distal symmetrical polyneuropathy (DSPN) and cardiovagal autonomic neuropathy (CAN) are two of the most frequent and relevant clinical manifestations of nerves’ involvement in diabetes [3]. Chronic exposure to hyperglycemia combined with oxidative and inflammatory stress are responsible for damage to peripheral nervous fibers [4]. DSPN can be distinguished by the caliber of the fibers involved. Damage to large sensory fibers manifests as numbness, painless paresthesias, and wool-like sensation, while damage to small fibers can lead to neuropathic pain (itching, burning sensation and dysesthesia as main symptoms) [3]. Pure large-fiber (LFN) and small-fiber (SFN) neuropathy are uncommon in diabetes, but mixed forms are frequent (up to 74% of cases) and neuropathic pain is similarly distributed in the small-fiber and mixed forms of neuropathy [5]. Previous studies reported that DSPN, especially when involving small fibers, may already be present in 10% to 30% of patients during the early phase of the disease, which is characterized primarily by impaired glucose tolerance (IGT) [6,7]. The diagnosis of DSPN is established by the exclusion of other potential causes, while the concomitant presence of diabetic nephropathy and/or retinopathy in the same patient increases the likelihood of DSPN. The use of diagnostic tools, such as the Michigan Neuropathy Screening Instrument (MNSI) questionnaire [8] and the Douleur Neuropathique 4 questionnaires (DN4) [9], is complemented by a comprehensive clinical evaluation (measurement of muscle force, ankle reflexes, vibratory perception threshold and 10 g monofilament sensitivity). Additionally, neurophysiological examinations are key to confirmation of polyneuropathy [4]. When symptoms caused by small-fiber damage, such as pain or hypoalgesia, together with impairment in thermal sensitivity, prevail and nerve conduction studies (NCS) are normal, a diagnosis of small-fiber neuropathy can be confirmed by other complementary tests [4]. The gold standard for definite diagnosis of SFN is skin biopsy, which assesses intraepidermal nerve fiber density (IENFd) [10]. However, current neurophysiological investigation of small-fiber damage relies on recording laser-evoked potentials (LEPs) [11], derived from laser-generated radiant heat pulses selectively stimulating small myelinated Aδ and unmyelinated C mechano-thermal nociceptors [12]. Despite their moderate sensitivity (78%) and specificity (81%), LEPs have been proposed as a less invasive alternative to skin biopsy for diagnosing diabetic small-fiber neuropathy [13]. Within the diagnostic work-up of small-fiber neuropathies, several alternative approaches include corneal confocal microscopy, microneurography, and blood-derived biomarkers. This reflects the growing interest in developing non- or minimally invasive methods for identifying neuropathy in diabetes [2,14]. CAN is caused by the damage of the small fibers of the autonomic nervous system responsible for modulating cardiac and vascular functions [15]. Its prevalence varies from 10 to 15% in IGT [16,17] to 65% in established type 2 diabetes mellitus (T2DM) [18]. Risk factors for autonomic dysfunction in diabetes include disease duration, glycemic control and variability, age, microvascular complications, hypertension, smoking, oxidative stress, and systemic inflammation [19,20]. CAN manifests with resting tachycardia and exercise intolerance. Early signs include abnormal heart rate variability (HRV) and parasympathetic dysfunction. Sympathetic involvement may lead to a reduced heart rate, while nearly complete cardiac denervation results in a fixed heart rate, unaltered by external factors. CAN also contributes to orthostatic hypotension, silent heart ischemia, and left ventricular diastolic dysfunction [18]. In fact, a recent investigation has elucidated the role of the adrenergic system in maintaining hemostatic blood pressure control, even during the prodromal stages of its functional impairment [21]. Clinical evaluation is mandatory in the diagnosis of CAN, together with cardiovascular autonomic reflex tests (CARTs) [15]. CARTs comprehend wide and standardized maneuvers to evaluate cardiovascular autonomic function by quantifying reflex changes in heart rate and blood pressure. The tests primarily assess vagal function through heart rate responses, and sympathetic function via blood pressure responses [22,23]. Although no universally accepted criteria for CAN diagnosis have been established to date, the Toronto Consensus Panel on the Cardiovascular Autonomic Neuropathy considers as the gold standard for diagnosing CAN the heart rate response to deep breathing, standing, and the Valsalva Maneuver (VM), together with the blood pressure response to standing [18].

In patients with diabetes, both DSPN affecting primarily the small fibers (i.e., painful neuropathy) and CAN can be simultaneously found [3], and some studies suggest that more severe levels of DPN are associated with worse outcomes in autonomic tests [24]. With the present study, we want to compare LEPs in a group of patients with T2DM and in healthy controls. Furthermore, given the limited evidence for a comparison between LEPs and CARTs, we searched for associations in the T2DM group and analyzed factors potentially associated with LEPs changes.

## 2. Materials and Methods

This study was conducted in accordance with STROBE guidelines for observational studies [25].

### 2.1. Patients

We retrospectively collected data from our medical records of male and female patients with diabetes (*n* = 33) who underwent both CARTs and LEPs at the Neurophysiological Center of our Institution between June 2023 and June 2024. All LEPs and autonomic reflex tests were performed after written informed consent was obtained. Inclusion criteria were T2DM; age < 75 years; symptomatic and asymptomatic illness. Exclusion criteria were inability to provide informed consent, major limb amputation, and other risk factors for neuropathy. The LEPs acquired from 33 patients without T2DM matched for age and sex served as controls. This study was conducted according to the principles of the Declaration of Helsinki.

### 2.2. Clinical and Laboratory Data

The T2DM group provided data on age, sex, diabetes duration, glycated hemoglobin (HbA1c), blood sugar, creatinine levels, and diabetes-related complications, such as renal failure, microalbuminuria, and retinopathy. Concomitant comorbidities such as heart failure and peripheral artery disease were also considered. The T2DM group completed the MNSI questionnaire [8] and underwent clinical examination (ankle reflexes, vibratory perception threshold, and 10 g monofilament sensitivity) to assess for an underlying DSPN. The DN4 questionnaire, Italian version, was administered to distinguish between nociceptive versus neuropathic pain, wherein a score of 4 or more was suggestive of neuropathic pain [9].

### 2.3. Evaluation of Cardiovascular Autonomic Reflex Tests (CARTs)

The T2DM group underwent comprehensive neurophysiological evaluation at the Neurophysiology Unit of Borgo Trento Hospital to assess potential impairment of the autonomic nervous system. They were instructed to refrain from engaging in any physical activity for at least 24 h prior to testing, and to avoid the consumption of caffeinated beverages or nicotine for at least 2 h before the examination. In addition, they had to discontinue taking α- or β- agonists/blockers, anticholinergics, sedatives or antihistamines for at least 24–48 h and any medication for neuropathic pain.

Variation in blood pressure and heart rate was evaluated via the “deep breathing” (DB) test, the VM and the “lying-to-standing” (LS) test, according to previously standardized methods [22,23]. We used a Cadwell Sierra^®^ Wave^®^ (version 9.0.309). The DB test result was quantified as the difference between the maximum and the minimum heart rates, derived by calculating the difference between the mean of the three highest recorded heart rates and the mean of the three lowest recorded heart rates, during 1 min of observation (consisting of six cycles, with each cycle comprising 5 s of inspiration followed by 5 s of expiration) [22]. The LS test was conducted to measure the change in blood pressure levels after changing from supine position to the standing position. The result is expressed as the ratio of the longest RR interval measured between the 20th and the 40th beat following postural change to the shortest RR interval measured between the 5th and the 25th beat [22]. The heart rate variation induced by pressure changes during the VM was quantified by measuring the Valsalva ratio (VR), which is derived from the ratio of the longest RR interval following exhalation to the shortest RR interval during exhalation [26]. Finally, the T2DM group underwent an orthostatic hypotension test, in which blood pressure was measured every 60 s in the upright position, with the arm kept horizontal at the level of the cardiac apex, until the fifth minute [22]. Autonomic cardiovagal dysfunction was diagnosed according to the criteria of Ewing and Clarke [27]. Dysfunction was classified as “uncertain” when only one test yielded abnormal results or when two tests were borderline, “confirmed” when two tests showed abnormal results, and “severe” when two such findings were present in a patient with orthostatic hypotension.

### 2.4. LEPs

As previously described [28,29], we used an Nd:YAP laser stimulator (Electronic Engineering, Florence, Italy) to record LEP response after hand and feet stimulation in the T2DM group and compared them against age-matched normative values. The T2DM group was asked to wear protective goggles and to lie down in a warm, semi-darkened room. Stimuli were delivered via a 5 mm laser beam lasting 5 ms in duration, 1.4 μm wavelength, and maximum energy of 6 J; the subjects were asked to rate the intensity of the stimulations to detect the threshold capable of activating Aδ fibers and to elicit a clear pinprick sensation. The threshold was defined as at least 4 on a numeric rating scale (NRS) from 0 to 10. The laser stimulus was delivered using ascending and descending energy series until the pain threshold was reached. The stimulus intensity was then set to 1.5 times the pain threshold and maintained constant during the exam. The subjects were told to relax their facial muscles, to keep their eyes open while fixing their gaze on the same point to minimize ocular artifact, and to mentally count the number of stimulations to maintain focus. The laser beam was moved to a different skin area to avoid skin irritation and nociceptor adaptation; the interstimulus interval (ISI) was between 20 and 30 s. LEPs were recorded using a Keypoint EMG (Dantec, Skovlunde, Denmark) by positioning surface Ag-AgCl electrodes on the frontal area (Fz) and the vertex area (Cz) referenced to the nose, and on the temporal area (T3 and T4) referenced to Fz, according to the 10–20 International System (recording parameters: amplification 30–50 µVolt/division, sweep length 200 msec/division, low filter 0.2 Hz, high filter 100 Hz). Eye movements were recorded by electrooculography (EOG) to discard ocular artifacts. LEP components were identified and marked according to Valeriani and coworkers [30]. Peak latency (Lat) and peak-to-peak amplitude (Amp) of the N2-P2 vertex complex were measured. Only the N2P2 response over the N1 was considered because of the good feasibility and reproducibility in our cohort, especially with foot dorsum stimulation. LEP acquisitions were performed by 3 trained operators (CB, MA, AS) and interpreted by an expert neurologist (GMS). LEPs were recorded bilaterally; since no difference between the two sides was found, only data from the right limbs were collected and analyzed in the two study groups.

### 2.5. Statistical Analysis

The Shapiro–Wilk test was used to check whether the variables had a Gaussian distribution. Cohen’s d was used to calculate effect size, with d < 0.5, 0.5 < d < 0.8, and d > 0.8 indicating a small, medium, and large effect, respectively. The results are expressed as the mean ± standard deviation (SD) for uniformity and graphical construction, regardless of the type of distribution; confidence intervals (CIs) for the relevant variables have been reported. T-test or Mann–Whitney U tests were used for two-group comparisons, while one-way ANOVA or Kruskal–Wallis tests with post hoc corrections were used for comparison for three groups or more. The chi-squared (χ^2^) test was employed for categorical variables. Linear correlations were assessed using Pearson’s or Spearman’s correlation coefficient to identify potential factors associated with LEPs; univariate and multivariate logistic regression analysis were performed, with the dependent variable being the amplitude of the N2-P2 component of the foot and hand, dichotomized on the basis of the 5th percentile of normative values. The T2DM group was divided into subgroups in order to analyze associations between clinical and laboratory variables and LEP latency and amplitude changes. For Hb1Ac, we use a threshold of 60 mmol/mol (a value close to the median distribution), while for disease duration, three subgroups (0–5 years, 6–10 years, >10 years) were created. Statistical significance was set at *p* < 0.05. Statistical analysis was performed using SPSS version 20 (IBM SPSS Statistics). The figures underwent graphical adjustment using ChatGPT (version 5.1) to improve visual clarity.

## 3. Results

There was no statistically significant difference in the male-to-female ratio (M/F 17/16 vs. 15/18, *p* = 0.81) or in the mean age (63.4 years vs. 64, *p* = 0.74; Table 1).

**Figure 1:**
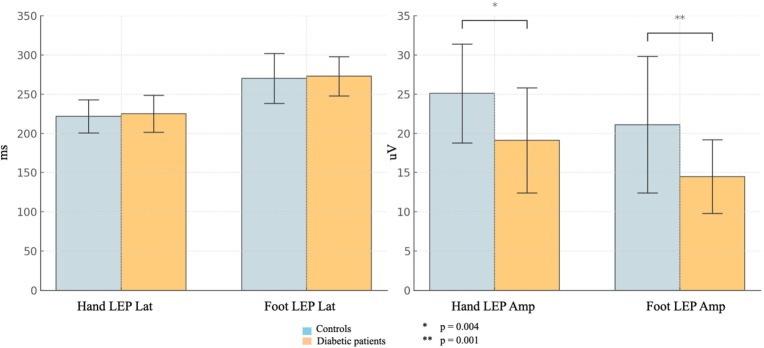
Comparison of LEP results in the two groups. Hand LEP Lat denotes LEP latency after right hand stimulation; Hand LEP Amp denotes LEP amplitude after right hand stimulation; Foot LEP Lat denotes LEP latency after right foot stimulation; Foot LEP Amp denotes LEP amplitude after right foot stimulation.

In the T2DM group, the mean N2/P2 Amp was lower for both the right hand and the right foot (hand: mean Amp 19.1 ± 6.7 [CI 16.7 uV–21.5 uV] vs. 25.1 ± 6.3 uV [CI 21 uV–27.5 uV], *p* = 0.004; foot: mean Amp 14.5 ± 4.7 uV [CI 12.9 uV–16.2 uV] vs. 21.1 ± 8.7 uV [CI 17.1 uV–24.2 uV], *p* = 0.001), while there was no significant difference in N2 Lat between the two groups (hand: mean Lat 224.9 ± 23.5 msec vs. 221.7 ±20.9 msec; foot Lat 272.8 ± 25.1 msec vs. 270 ±31.7 msec). Cohen’s d using LEP amplitude was 0.92 and 0.94, respectively, from right hand and right foot, thus indicating a large effect size (Figure 1).

There was a statistical trend for longer disease duration, higher blood glucose, and HbA1c values, albeit not significant (mean duration, 14.2 vs. 11.06 years, HbA1c values, 53.8 vs. 57.1 mm/L, blood sugar levels, 131.2 vs. 138.9 mg/dL) according to the 5th percentile of normative values of N2/P2 amplitude after right hand (16.9 uV) and foot stimulation (12.4 uV).

**Figure 2:**
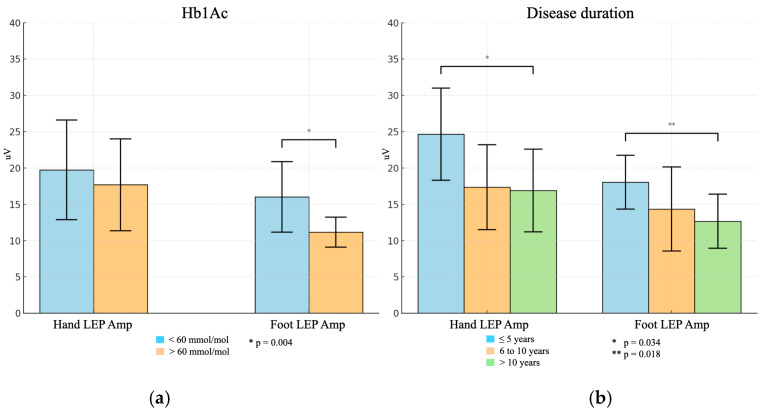
This figure represents the results of LEPs in the T2DM group subdivided by the Hb1Ac threshold of 60 mmol/mol (**a**) and disease duration (**b**). Hand LEP Lat denotes LEP latency after right hand stimulation; Hand LEP Amp denotes LEP amplitude after right hand stimulation; Foot LEP Lat denotes LEP latency after right foot stimulation; Foot LEP Amp denotes LEP amplitude after right foot stimulation.

In the T2DM group, LEP hand Lat was strongly correlated with foot LEP Lat (Rho 0.736, *p* < 0.001) and foot LEP Amp was moderately related to hand LEP Amp (Rho 0.511, *p* = 0.002). Hand LEP Lat and foot LEP Lat were inversely correlated with hand and foot LEP Amp (Rho −0.518, *p* = 0.002 and Rho −0.547, *p* = 0.001, respectively). There was a moderate correlation between HbA1c level and disease duration (Rho 0.402, *p* = 0.021) and HbA1c level and blood glucose (Rho 0.540, *p* = 0.001). There was an inverse association between HbA1c level and foot N2/P2 amplitude (Rho 0.410, *p* = 0.018), which was confirmed when the LEP results were compared with glycemic control (HbA1c <60 mmol/mol vs. HbA1c >60 mmol/mol). For this latter analysis, two subgroups were created with 23 patients below the threshold of 60 mmol/mol and 10 patients above that limit. There were differences in LEPs between the two groups: N2 latency right hand 223.78 ± 20.68 vs. 227.50 ± 30.18; N2/P2 amplitude right hand 19.75 ± 6.86 vs. 17.70 ± 6.33; N2 latency right foot 270.70 ± 28.29 vs. 277.60 ± 15.91; N2/P2 right foot 16.02 ± 4.86 vs. 11.17 ± 2.07. A statistically significant difference was noted only for N2/P2 amplitude derived from right foot stimulation (*p* = 0.004, Figure 2, Table 2).

Disease duration was related to hand LEP Lat (Rho 0.355, *p* = 0.043) and foot LEP lat (Rho 0.359, *p* = 0.040) and conversely associated with hand LEP Amp (Rho −0.407; *p* = 0.019) and foot LEP Amp (Rho −0.494, *p* = 0.003). Division into three subgroups according to duration of disease (≤5 vs. from 6 to 10 vs. >10 years) yielded differences (N2 latency right hand: 213.00 ± 15.06 vs. 225.15 ± 28.08 vs. 231.50 ± 23.66; N2/P2 amplitude right hand: 24.66 ± 6.34 vs. 17.35 ± 5.85 vs. 16.91 ± 5.69; N2 latency right foot: 259.78 ± 20.34 vs. 273.00 ± 23.89 vs. 280.00 ± 26.51; N2/P2 amplitude right foot: 18.06 ± 3.70 vs. 14.36 ± 5.79 vs. 12.67 ± 3.73), with a statistically significant difference for LEPs Amp, for both right hand and foot stimulation (*p* = 0.028 and *p* = 0.018, respectively) (Figure 2 and Table 2). There was a statistically significant difference between the first (≤5 years of disease duration) and the third group (>10 years of disease duration) (*p* = 0.034 considering the hand LEP Amp and *p* = 0.018 for foot LEP Amp) in the three subgroups (9, 8, and 16 patients, respectively) (Figure 2).

The results of the cardiovascular autonomic reflex tests (CARTs), according to Ewing and Clarke’s criteria [27], showed that 49% of our cohort did not present involvement of the autonomic nervous system, 51% fit the criteria for “uncertain”, and only one patient was classified as “confirmed”.

The χ^2^ test showed that the CART parameters were not statistically correlated with LEP results (both for latency and amplitude derived from the right hand and foot). However, abnormal LEPs (values above the 5th percentile of normative values) were observed in T2DM patients with a nearly significant correlation with the VR (*p* = 0.07). Moreover, there were moderate correlations between LS and DB (Rho 0.353, *p* = 0.044) and VR (Rho 0.418, *p* = 0.022). The VR was also correlated with disease duration (Rho 0.413, *p* = 0.023).

## 4. Discussion

In this study, we found a significantly reduced mean amplitude of LEPs after hand and foot stimulation in the T2DM versus the healthy control group. The discrepancy was more pronounced in the lower limbs, possibly reflecting nociceptor loss secondary to a length-dependent mechanism of disease. Moreover, we observed a correlation between hand LEP Lat and foot LEP Lat and between hand LEP Amp and foot LEP Amp, not seen in the normal controls, which might be the expression of a homogeneous damage along nerve course. Previous studies [31,32] found that, in patients with diabetes and LFN, slowed motor conduction was not primarily caused by loss of large axons; rather, there was an additional multifocal demyelinating component in intermediate nerve segments. Conversely, our findings demonstrated an inverse correlation between LEP amplitude and LEP latency, which may suggest a slowing of conduction proportional to axonopathy, thus strengthening the long-standing controversy over the pathophysiology of diabetic neuropathy [33]. These findings support LEPs as a sensitive biomarker for early small-fiber neuropathy in diabetes, in agreement with previous studies [12,34,35,36]. However, those studies [34,35,36] reported significant differences only in the LEPs acquired from foot stimulation, while we recorded a significantly decreased N2/P2 amplitude in the LEPs recorded from hand stimulation as well. A possible explanation for this discrepancy is the different inclusion criteria we adopted, given that, in our study, we also enrolled patients with clinical evidence of DSPN, while the authors included asymptomatic patients or diabetics with negative clinical examination or normal nerve conduction studies. Furthermore, unlike other authors [35,36], and in agreement with Ragè [34] and Agostino [37], we found alterations only in N2/P2 amplitude, while the latency increase did not reach statistical significance; this finding can be explained by the fact that in DSPN, the damage is more frequently axonopathic (thus, compromising N2/P2 amplitude in the first place) and length-dependent (primarily affecting foot fibers) [37].

We report lower foot amplitudes associated with longer diabetes duration and higher HbA1c levels. Notably, a progressive decrease in N2/P2 foot amplitude was observed even in early-stage diabetes, suggesting time-dependent small-fiber impairment. Both disease duration and higher HbA1c levels are well-known risk factors for the development of diabetic neuropathy [3] and were found to correlate with reduced N2/P2 amplitude in our study. This observation corroborates previous findings [35] and reinforces the hypothesis that cumulative metabolic exposure to hyperglycemia plays a pivotal role in the pathogenesis of small-fiber dysfunction. Importantly, our data demonstrate a significant reduction in N2/P2 amplitude in the first years following diabetes onset, suggesting that LEPs may detect subclinical and temporally early involvement of nociceptive small fibers. This is particularly relevant considering that T2DM may remain asymptomatic for 4 to 7 years prior to clinical diagnosis [38], further supporting the utility of LEPs as a sensitive biomarker for early neuroaxonal impairment in this population.

The current gold-standard diagnostic tests for small-fiber neuropathy are the Quantitative Sensory Testing (QST) and skin biopsy to assess IENFd [11,39]. Previous studies have demonstrated a correlation between IENFd and Aδ-fiber-related evoked potentials in peripheral neuropathy [40]. These findings are consistent with those reported by Ragè and coworkers [34], who demonstrated that LEP results correlated with IENFd in asymptomatic diabetic SFN with a sensitivity of 91% and a specificity of 83% for detecting small-fiber loss. Similarly, Di Stefano and collaborators [13] reported LEP sensitivity and specificity of 78% and 81%, respectively, in identifying small-fiber damage. Within this context, the reduction in N2/P2 amplitude we observed may be interpreted as a reliable neurophysiological marker of small nociceptive fiber impairment. This observation gains further significance considering that only two patients in our cohort had a positive DN4 questionnaire, underscoring the potential utility of this marker for the early detection of nociceptive fiber involvement. Furthermore, skin biopsy is an invasive procedure and is frequently employed as a second-line diagnostic test in routine clinical practice. Accordingly, at the time of our retrospective analysis, none of the patients in our cohort had undergone this investigation.

Unexpectedly, unlike other reports [34], we found no correlation between LEP alterations and microangiopathies or cardiovascular comorbidities; we interpreted this fact as probably due to the small sample size (i.e., only two patients had proliferative diabetic retinopathy).

Since SFN may manifest on a continuum of DSPN and CAN [41], we searched for a possible correlation between these two conditions but could find none (abnormal LEP results were observed to have only a near-significant correlation with VR), similarly to a previous study by Pozzerese and colleagues [35], where autonomic disfunction was evaluated with a pupillometric analysis. Laser stimulation is known to activate both Aδ and C nociceptive fibers, but since the cortical responses elicited by our type of stimulation reflect the activation of Aδ fibers [42], it may be postulated that the absence of LEP abnormalities in our cohort with CAN suggests a predominant or exclusive impairment of C fibers. Alternatively, this dissociation may indicate that autonomic and somatosensory small-fiber subtypes exhibit differential vulnerability or follow distinct pathogenic trajectories in diabetic neuropathy. Such assumptions remain speculative given the small sample size and the retrospective design of our study, and they reflect the preferential investigation of Aδ fibers via LEPs. To the best of our knowledge, only Pozzessere and coworkers [35] have sought to examine the possible relationship between CAN and abnormalities in LEPs. Instead, we observed correlations between the VR and the LS test and between the VR and disease duration, which indicate progressive autonomic nervous system involvement during the disease course and confirm published data reporting an ongoing development of CAN with disease progression [19]. The VR is one of the most sensitive indices to detect autonomic abnormalities even in the initial phase of diabetes [26]; therefore, our findings align with previous clinical evidence and further substantiate the reliability of this parameter as a clinically meaningful marker for assessing potential autonomic nervous system involvement in patients with diabetes.

Our study addresses the early detection of small-fiber neuropathy in patients with T2DM (both symptomatic and asymptomatic) and shows how non-invasive techniques may be useful in reaching an earlier diagnosis [14]. Malik [43] and Ragè [34] reported the urgent need for updating our screening tests for diabetic neuropathy since the 10 g monofilament test only identifies patients with advanced neuropathy. This need becomes even more compelling when we remember that the 5-year mortality of a diabetic patient with a foot ulcer is higher than with most cancers [43], and growing evidence suggest that lifestyle modification can significantly impact the progression of neuropathic involvement [44]. The published data [45] show that the acquisition of LEPs is an easy-to-perform, non-invasive test that could help detect early and asymptomatic small-fiber damage [14]. Unlike skin biopsy, LEPs can reliably identify nociceptive fiber dysfunction and assess A-δ fibers [7]. Moreover, IENFd correlates with A-δ fiber-related evoked potentials. In conclusion, there is a growing body of evidence suggesting that evaluation of LEPs, together with other tests of small-fiber damage (such as microneurography and corneal confocal microscopy), could enable timely intervention and give an early go–no-go signal in clinical trials of disease-modifying therapies in diabetic neuropathy [43].

Finally, our study has two limitations: the retrospective design of this study and the relatively small sample size, with a small number of patients within each subgroup. These limitations notwithstanding, the patients were well-characterized and LEP acquisition was conducted by the same operators and interpreted by the same neurologist to enhance reliability.

## 5. Conclusions

This study presents findings for LEPs as an early biomarker of small-fiber involvement in T2DM patients and regarding the association between neuropathy, diabetes duration, and poor metabolic control. There is an urgent need to revise routinary tests for neuropathy screening in diabetes care to detect early alterations and, possibly, modify the disease course. Despite the hypothesis for a shared pathophysiological mechanism, we found no correlation between LEP alteration and CARTs, possibly because of the different type of small fibers studied with these techniques. Further studies with larger patient samples would be advisable to validate our results.

## Figures and Tables

**Table 1:** Study groups divided by age and by sex.

		T2DM (*n* = 33)	Controls (*n* = 33)	*p*
Age (y)		64.0 ± 6.9	63.4 ± 7.7	0.74
Sex (M/F)		17/16	15/18	0.81
LEP (right hand)	N2 latency (ms)	224.91 ± 23.52 (CI 216.57–233.25)	221.7 ± 20.9 (CI 215.67–239.2)	>0.05
	N2/P2 amplitude (uV)	19.13 ± 6.7 (CI 16.7 uV–21.5 uV)	25.1 ± 6.3 (CI 21–27.5)	0.004
LEP (right foot)	N2 latency (ms)	272.79 ± 25.14 (CI 263.87–281.7)	270 ± 31.7 (CI 250.5–281.62)	>0.05
	N2/P2 amplitude (uV)	14.5 ± 4.7 (CI 12.9–16.2)	21.1 ± 8.7 (CI 17.1–24.2)	0.001

**Table 2:** The table presents the LEP results of the T2DM subgroup divided according to Hb1Ac and disease duration. Significant results were noted for LEPs derived from the right foot (for both sub-analyses) and from the right hand (only for the disease duration analysis).

	LEP (Right Hand)		LEP (Right Foot)	
Characteristic	N2 latency (ms)	N2/P2 amplitude (uV)	N2 latency (ms)	N2/P2 amplitude (uV)
HbA1c > 60 mmol/L	227.50 ± 30.18	17.70 ± 6.33	277.60 ± 15.91	11.17 ± 2.07
HbA1c < 60 mmol/L	223.78 ± 20.68	19.75 ± 6.86	270.70 ± 28.29	16.02 ± 4.86
	*p* > 0.05	*p* > 0.05	*p* > 0.05	*p* = 0.004
Disease duration ≤ 5 years	213.00 ± 15.06	24.66 ± 6.34	259.78 ± 20.34	18.06 ± 3.70
Disease duration from 6 to 10 years	225.15 ± 28.08	17.35 ± 5.85	273.00 ± 23.89	14.36 ± 5.79
Disease duration > 10 years	231.50 ± 23.66	16.91 ± 5.69	280.00 ± 26.51	12.67 ± 3.73
	*p* > 0.05	*p* = 0.028	*p* > 0.05	*p* = 0.018

## Data Availability

Regarding privacy restrictions, we would like to clarify that any complete statistical analyses or clinical, laboratory or neurophysiological data are available only upon request to the corresponding author.

## Abbreviations

The following abbreviations are used in this manuscript:

CARTs	Cardiovascular autonomic reflex tests
LEPs	Laser-evoked potentials
DSPN	Distal symmetrical polyneuropathy
CAN	Cardiovagal autonomic neuropathy
LFN	Large-fiber neuropathy
SFN	Small-fiber neuropathy
MNSI	Michigan Neuropathy Screening Instrument
DN4	Douleur Neuropathique 4 questionnaires
T2DM	Type 2 diabetes mellitus
HbA1c	Glycated hemoglobin
DB	Deep breathing
LS	Lying to standing
VM	Valsalva maneuver
IENFd	Intraepidermal nerve fiber density
IGT	Impaired glucose tolerance
CI	Confidence interval

## References

[1] Albers J.W., Pop-Busui R. (2014). Diabetic neuropathy: Mechanisms, emerging treatments, and subtypes. Curr. Neurol. Neurosci. Rep..

[2] Al-Khafaji M., Otti V. (2025). Early Diagnosis of Diabetic Neuropathy: A Review of Current Diagnostic Tests. Cureus.

[3] Pop-Busui R., Boulton A.J.M., Feldman E.L., Bril V., Freeman R., Malik R.A., Sosenko J.M., Ziegler D. (2017). Diabetic Neuropathy: A Position Statement by the American Diabetes Association. Diabetes Care.

[4] Tesfaye S., Boulton A.J.M., Dyck P.J., Freeman R., Horowitz M., Kempler P., Lauria G., Malik R.A., Spallone V., Vinik A. (2010). Diabetic neuropathies: Update on definitions, diagnostic criteria, estimation of severity, and treatments. Diabetes Care.

[5] Galosi E., Di Pietro G., La Cesa S., Di Stefano G., Leone C., Fasolino A., Di Lionardo A., Leonetti F., Buzzetti R., Mollica C. (2021). Differential involvement of myelinated and unmyelinated nerve fibers in painful diabetic polyneuropathy. Muscle Nerve.

[6] Callaghan B.C., Xia R., Banerjee M., de Rekeneire N., Harris T.B., Newman A.B., Satterfield S., Schwartz A.V., Vinik A.I., Feldman E.L. (2016). Metabolic Syndrome Components Are Associated With Symptomatic Polyneuropathy Independent of Glycemic Status. Diabetes Care.

[7] Singleton J.R., Smith A.G., Bromberg M.B. (2001). Increased prevalence of impaired glucose tolerance in patients with painful sensory neuropathy. Diabetes Care.

[8] Moghtaderi A., Bakhshipour A., Rashidi H. (2006). Validation of Michigan neuropathy screening instrument for diabetic peripheral neuropathy. Clin. Neurol. Neurosurg..

[9] Bouhassira D., Attal N., Alchaar H., Boureau F., Brochet B., Bruxelle J., Cunin G., Fermanian J., Ginies P., Grun-Overdyking A. (2005). Comparison of pain syndromes associated with nervous or somatic lesions and development of a new neuropathic pain diagnostic questionnaire (DN4). Pain.

[10] Malik R.A., Veves A., Tesfaye S., Smith G., Cameron N., Zochodne D., Lauria G. (2011). Small fibre neuropathy: Role in the diagnosis of diabetic sensorimotor polyneuropathy. Diabetes Metab. Res. Rev..

[11] Marshall A., Alam U., Themistocleous A., Calcutt N., Marshall A. (2021). Novel and Emerging Electrophysiological Biomarkers of Diabetic Neuropathy and Painful Diabetic Neuropathy. Clin. Ther..

[12] Cruccu G., Aminoff M.J., Curio G., Guerit J.M., Kakigi R., Mauguiere F., Rossini P.M., Treede R.D., Garcia-Larrea L. (2008). Recommendations for the clinical use of somatosensory-evoked potentials. Clin. Neurophysiol..

[13] Di Stefano G., La Cesa S., Leone C., Pepe A., Galosi E., Fiorelli M., Valeriani M., Lacerenza M., Pergolini M., Biasiotta A. (2017). Diagnostic accuracy of laser-evoked potentials in diabetic neuropathy. Pain.

[14] Røikjer J., Borbjerg M.K., Andresen T., Giordano R., Hviid C.V.B., Mørch C.D., Karlsson P., Klonoff D.C., Arendt-Nielsen L., Ejskjaer N. (2024). Diabetic Peripheral Neuropathy: Emerging Treatments of Neuropathic Pain and Novel Diagnostic Methods. J. Diabetes Sci. Technol..

[15] Spallone V. (2019). Update on the Impact, Diagnosis and Management of Cardiovascular Autonomic Neuropathy in Diabetes: What Is Defined, What Is New, and What Is Unmet. Diabetes Metab. J..

[16] Ziegler D., Voss A., Rathmann W., Strom A., Perz S., Roden M., Peters A., Meisinger C. (2015). Increased prevalence of cardiac autonomic dysfunction at different degrees of glucose intolerance in the general population: The KORA S4 survey. Diabetologia.

[17] Zoppini G., Cacciatori V., Raimondo D., Gemma M., Trombetta M., Dauriz M., Brangani C., Pichiri I., Negri C., Stoico V. (2015). Prevalence of Cardiovascular Autonomic Neuropathy in a Cohort of Patients With Newly Diagnosed Type 2 Diabetes: The Verona Newly Diagnosed Type 2 Diabetes Study (VNDS). Diabetes Care.

[18] Spallone V., Ziegler D., Freeman R., Bernardi L., Frontoni S., Pop-Busui R., Stevens M., Kempler P., Hilsted J., Tesfaye S. (2011). Cardiovascular autonomic neuropathy in diabetes: Clinical impact, assessment, diagnosis, and management. Diabetes Metab. Res. Rev..

[19] Kalopita S., Liatis S., Thomakos P., Vlahodimitris I., Stathi C., Katsilambros N., Tentolouris N., Makrilakis K. (2014). Relationship between autonomic nervous system function and continuous interstitial glucose measurement in patients with type 2 diabetes. J. Diabetes Res..

[20] Serhiyenko V.A., Serhiyenko A.A. (2018). Cardiac autonomic neuropathy: Risk factors, diagnosis and treatment. World J. Diabetes.

[21] Souza G.M.P.R., Thakkalapally H., Berry F.E., Atongazi U.M., Stornetta D.S., Abbott S.B.G. (2026). Control of Blood Pressure Variability Across Behavioral States by Brainstem Adrenergic Neurons. Circ. Res..

[22] Freeman R. (2006). Assessment of cardiovascular autonomic function. Clin. Neurophysiol..

[23] Hilz M.J., Dütsch M. (2006). Quantitative studies of autonomic function. Muscle Nerve.

[24] de Paula A.V.L., Dykstra G.M., da Rocha R.B., Magalhães A.T., da Silva B.A.K., Cardoso V.S. (2024). The association of diabetic peripheral neuropathy with cardiac autonomic neuropathy in individuals with diabetes mellitus: A systematic review. J. Diabetes Complicat..

[25] von Elm E., Altman D.G., Egger M., Pocock S.J., Gøtzsche P.C., Vandenbroucke J.P. (2008). Declaración de la Iniciativa STROBE (Strengthening the Reporting of Observational studies in Epidemiology): Directrices para la comunicación de estudios observacionales. Gac. Sanit..

[26] Ziegler D., Dannehl K., Volksw D., Mühlen H., Spüler M., Gries F.A. (1992). Prevalence of cardiovascular autonomic dysfunction assessed by spectral analysis and standard tests of heart-rate variation in newly diagnosed IDDM patients. Diabetes Care.

[27] Ewing D.J., Clarke B.F. (1986). Autonomic neuropathy: Its diagnosis and prognosis. Clin. Endocrinol. Metab..

[28] Squintani G., Brugnoli M.P., Pasin E., Segatti A., Concon E., Polati E., Bonetti B., Matinella A. (2018). Changes in laser-evoked potentials during hypnotic analgesia for chronic pain: A pilot study. Ann. Palliat. Med..

[29] Turri M., Teatini F., Donato F., Zanette G., Tugnoli V., Deotto L., Bonetti B., Squintani G. (2018). Pain Modulation after Oromucosal Cannabinoid Spray (SATIVEX^®^) in Patients with Multiple Sclerosis: A Study with Quantitative Sensory Testing and Laser-Evoked Potentials. Medicines.

[30] Valeriani M., Rambaud L., Mauguière F. (1996). Scalp topography and dipolar source modelling of potentials evoked by CO2 laser stimulation of the hand. Electroencephalogr. Clin. Neurophysiol..

[31] Wilson J.R., Stittsworth J.D., Kadir A., Fisher M.A. (1998). Conduction velocity versus amplitude analysis: Evidence for demyelination in diabetic neuropathy. Muscle Nerve.

[32] Herrmann D.N., Ferguson M.L., Logigian E.L. (2002). Conduction slowing in diabetic distal polyneuropathy. Muscle Nerve.

[33] Behse F., Buchthal F., Carlsen F. (1977). Nerve biopsy and conduction studies in diabetic neuropathy. J. Neurol. Neurosurg. Psychiatry.

[34] Ragé M., Van Acker N., Knaapen M.W.M., Timmers M., Streffer J., Hermans M.P., Sindic C., Meert T., Plaghki L. (2011). Asymptomatic small fiber neuropathy in diabetes mellitus: Investigations with intraepidermal nerve fiber density, quantitative sensory testing and laser-evoked potentials. J. Neurol..

[35] Pozzessere G., Rossi P., Gabriele A., Cipriani R., Morocutti A., Di Mario U., Morano S. (2002). Early detection of small-fiber neuropathy in diabetes: A laser-induced pain somatosensory-evoked potentials and pupillometric study. Diabetes Care.

[36] Rossi P., Morano S., Serrao M., Gabriele A., Di Mario U., Morocutti C., Pozzessere G. (2002). Pre-perceptual pain sensory responses (N1 component) in type 1 diabetes mellitus. Neuroreport.

[37] Agostino R., Cruccu G., Romaniello A., Innocenti P., Inghilleri M., Manfredi M. (2000). Dysfunction of small myelinated afferents in diabetic polyneuropathy, as assessed by laser evoked potentials. Clin. Neurophysiol..

[38] Harris M.I., Klein R., Welborn T.A., Knuiman M.W. (1992). Onset of NIDDM occurs at least 4-7 yr before clinical diagnosis. Diabetes Care.

[39] Devigili G., Lombardi R., Lauria G., Cazzato D. (2025). The Evolving Landscape of Small Fiber Neuropathy. Semin. Neurol..

[40] Casanova-Molla J., Grau-Junyent J.M., Morales M., Valls-Solé J. (2011). On the relationship between nociceptive evoked potentials and intraepidermal nerve fiber density in painful sensory polyneuropathies. Pain.

[41] Basantsova N.Y., Starshinova A.A., Dori A., Zinchenko Y.S., Yablonskiy P.K., Shoenfeld Y. (2019). Small-fiber neuropathy definition, diagnosis, and treatment. Neurol. Sci..

[42] Valeriani M., Pazzaglia C., Cruccu G., Truini A. (2012). Clinical usefulness of laser evoked potentials. Neurophysiol. Clin..

[43] Malik R.A. (2016). Wherefore Art Thou, O Treatment for Diabetic Neuropathy?. Int. Rev. Neurobiol..

[44] Stirane L., Stirans K., Pahirko L., Mednieks J., Ostrovskas K., Klavina A., Selavo L., Sokolovska J. (2025). Assessing the impact of supervised interval training on cardiovascular autonomic neuropathy in type 2 diabetes patients. Physiol. Rep..

[45] Drobinska N., Nehme M., Assal F., Laffitte E., Guessous I., Lascano A.M. (2025). Small Fiber Neuropathy in Long COVID: A Cohort Study with Multimodal Assessment and Follow-Up. Eur. Neurol..

